# Computationally Efficient Continuous-Time Model Predictive Control of a 2-DOF Helicopter via B-Spline Parameterization

**DOI:** 10.3390/s23094463

**Published:** 2023-05-03

**Authors:** Boris Rohaľ-Ilkiv, Martin Gulan, Peter Minarčík

**Affiliations:** 1 Institute of Automation, Measurement and Applied Informatics, Faculty of Mechanical Engineering, Slovak University of Technology in Bratislava, 812 31 Bratislava, Slovakia; 2 Prosystemy, s.r.o. (Ltd.), 900 86 Budmerice, Slovakia

**Keywords:** continuous-time model predictive control, spline functions, 2-DOF helicopter, adaptive control

## Abstract

This paper investigates one way to reduce the computational burden of continuous-time model predictive control (MPC) laws by representing the input/output signals and related models using B-spline functions. Such an approximation allows to implement the resulting feedback control law more efficiently, requiring less online computational effort. As a result, the proposed controller formulates the control signals as continuous polynomial spline functions. All constraints assumed over the prediction horizon are then expressed as constraints acting on the B-splines control polygon vertices. The performance of the proposed theoretical framework has been demonstrated with several real-time experiments using the well-known 2-DOF laboratory helicopter setup. The aim of the presented experiments was to track given step-like reference trajectories for pitch and yaw angles under notable parameter uncertainties. In order to suppress the influence of uncertainties, the control algorithm is implemented in an adaptive mode, equipped with the recursive least squares (RLS) estimation of model parameters and with the adaptation of stabilizing terminal set and terminal cost calculations. Thanks to the presented framework, it is possible to significantly reduce the computational burden, measured by the number of decision variables and input constrains, indicating the potential of the proposed concept for real-time applications, even when using embedded control hardware.

## 1. Introduction

The notion of spline has become an important and well-established tool in the modern approximation theory. At present, splines are successfully applied in an increasing number of scientific and technical disciplines, as can be seen, e.g., in [1,2,3]. Splines are, roughly speaking, functions which serve the purpose of approximating or modeling other functions or discrete data. Originally, splines were defined by I.J. Schoenberg in 1946 as univariate piecewise polynomial functions. One of the fundamental contributions to the theory of polynomial splines represents the discovery of compactly supported basis functions, the so-called B-splines, by Curry and Schoenberg in 1966. B-splines are piecewise polynomial functions which form a basis for the space of polynomial splines. Their importance arises from the fact that their properties make them highly suitable for practical computations. Being polynomial, they can be evaluated very quickly; being piecewise polynomial, they are very flexible. The number and profile of the B-spline functions is clearly determined by the distribution of the so-called knot points and it is possible to change their behavior by changing the position of these knots. The ability of splines to achieve a very high quality of approximation in various technical applications inspired the authors to use them when looking for alternative ways to reduce the computational complexity of continuous-time MPC laws.

For studying and the real-time testing of the performance of various control techniques and strategies, 2-DOF laboratory helicopters of different constructions and designs have become increasingly popular platforms, see, e.g., [4,5,6,7]. These physical models represent typically nonlinear mechatronic systems with strong cross-couplings and parameters uncertainties which make control of the systems much more complicated. Moreover, according to the design, these systems can have time-varying dynamics and some parameters have to be estimated online using, e.g., recursive least squares. In this article, using such a laboratory system, we will investigate the computational efficiency of the B-spline parameterization technique in designing a practical continuous-time model predictive control (CMPC) procedure. The techniques for the efficient design and implementation of CMPC schemes are the subject of systematic research, motivated by many advantages that these techniques offer compared with common discrete-time MPC formulations. Some well-known schemes for designing such CMPC strategies can be found, for example, in [8,9,10,11,12,13], where different ways to preserve the time-continuity properties of the design and its independence on the chosen sampling rate are discussed.

In the works by [14,15,16,17], the authors of this paper have proposed an alternative approach to the CMPC problem solution based on B-spline functions parameterization. It can be shown that if we assume a finite number of B-spline basis functions, we may appropriately parameterize the control signal as a continuous spline curve with a predefined degree of continuity. The solution can provide some new benefits in CMPC problem formulation, such as the intersample satisfaction of constraints and the formulation of smooth control signals. In this procedure, the user selects the orders of the splines used to approximate the input and output signals in the prediction horizon and also determines the number and distribution of their knot points. The appropriate selection of these parameters thus significantly reduces the dimensionality of the corresponding optimization problem, i.e., the length of the optimizer, which becomes the control polygon of B-spline functions. The advantage of the spline formulation of the CMPC is also the possibility to preserve the original continuous boundaries appearing in the optimization problem by simply rewriting them to the boundaries of the elements of the control polygon.

In predictive control applications, the ability of the control law to follow predefined reference values is very often required. Possible approaches to solving this problem for the usual discrete-time MPC formulations are discussed, e.g., in [18,19,20]. As a novelty in this article, the authors present an extension of the spline velocity version of the CMPC algorithms, cited in their previous works [15,16,17], to multivariable nonlinear systems with an adaptation of the model parameters. The intention here is to achieve offset-free tracking of a set of piecewise-constant reference values in the presence of random disturbances. In addition, for the first time, we present the real-time deployment of the proposed algorithm to control a real-world system—a 2-DOF laboratory helicopter.

As already mentioned, these laboratory systems, same as the real-world helicopters, are in fact nonlinear and nonstationary systems, with their properties changing with the operating point over trajectory and time. These changes can be so significant that fixed feedback control may be unacceptable or even impossible. The effort to employ more advanced controllers—capable of adapting their setup according to the changing conditions or effects of random disturbances—is therefore a subject of constant interest in practice. Because we do not consider a more complex, nonlinear model of the controlled system, the adaptive approach appears as a feasible and simple solution. In terms of control, the explicit solution approaches to CMPC proposed by the authors in [16,17] seek an efficient representation of the predictive control law which, however, in implementation stays fixed, i.e., time-invariant. This paper therefore puts forward an implicit form of the spline-based CMPC in an adaptive mode, i.e., with adaptive parameter estimation, using the recursive identification and adaptive calculation of a stabilizing terminal set at each sampling instant.

In this work, the helicopter is modeled online using the RLS library [21]. The resulting helicopter multi-input multi-output (MIMO) model is created using the well-known technique of multi-input single-output (MISO) models [22]. In view of the real-time implementation, the developed adaptive B-spline-based CMPC controllers were deployed by means of the MATLAB/Simulink Desktop Real-Time software environment.

## 2. Spline and B-Spline Functions

In this section, we provide a brief summary of the basic properties of B-spline functions, conversions between B- and pp-spline representations, as well as their essential shape properties. For a comprehensive theory of splines, we refer the interested reader to [23,24,25,26]. In terms of content, the following mathematical background is mainly recalled from [16].

### 2.1. Basic Definitions

Let us first state some formal definitions.

**Definition 1.**  *Let $\left(\xi_{0}=\right)0<\xi_{1}<\ldots <\xi_{q}<T_{x}\left(=\xi_{q+1}\right)$ be the subdivision of a closed finite-time interval $\left[0,T_{x}\right]$ by q distinct (time) points. A function s(t), defined on the interval $\left[0,T_{x}\right]$, is called a spline function of the order r>0 (degree r−1) and the defect $d_{\mathrm{ef}}$ if the following two conditions hold:*

- *In each open interval $]\xi_{i},\xi_{i+1}[,i=0,\ldots ,q,$s(t) is a polynomial of degree ≤r−1;*
- *It has continuous derivatives up to the order $r-d_{\mathrm{ef}}-1$ in the open interval $]0,T_{x}[$.*

The points $\xi_{i},i=1,\ldots ,q$ are referred to as interior knots or breakpoints of the spline function. For each fixed set $\xi =(\xi_{1},\ldots \xi_{q})$ of the knots, the class of splines is a linear space of functions with dimension

(1) $$z=(\alpha_{1}+\alpha_{2}+\ldots +\alpha_{q})+r,$$

where $\alpha_{i}$ denotes a multiplicity (or defect $d_{\mathrm{ef}}$) of the knot $\xi_{i}$. Let $P_{r,\xi ,\alpha }$ denote the linear space of spline functions for $\alpha =(\alpha_{1},\ldots \alpha_{q})$. If all interior knots, $\xi_{i}$, are simple, i.e., $\alpha_{i}=1,i=1,\ldots ,q$, then $P_{r,\xi ,\alpha }=C^{r-2}\left[0,T_{x}\right]$. In order to perform computations with splines, one must first choose a suitable representation, in which any member of $P_{r,\xi ,\alpha }$ can be written as a unique linear combination of properly chosen *z* basis functions such that Definition 1 is satisfied. A common choice is to use B-spline functions.

**Definition 2.**  *A B-spline function $N_{i,r,\xi }\left(t\right)$ of order r>0, with knots $\xi_{i},\ldots ,\xi_{i+r},$ can be defined using the following recurrence relation:*

$$\begin{array}{cc} N_{i,1,\xi }\left(t\right) & :=\left\{\begin{array}{cc} 1 & if\;\xi_{i}\leq t<\xi_{i+1},\\ 0 & otherwise, \end{array}\right.\\ N_{i,k,\xi }\left(t\right) & :=\frac{\xi_{i+k}-t}{\xi_{i+k}-\xi_{i+1}}N_{i+1,k-1,\xi }\left(t\right)+\frac{t-\xi_{i}}{\xi_{i+k-1}-\xi_{i}}N_{i,k-1,\xi }\left(t\right),\;\;\mathrm{for}\;k=2,\ldots ,r, \end{array}$$

*where the two fraction terms are interpreted as zero whenever $\xi_{i+k}-\xi_{i+1}=0$ and $\xi_{i+k-1}-\xi_{i}=0$, respectively.*

From Definition 2, one can observe that $N_{i,r,\xi }\left(t\right),i=\ldots 0,1\ldots$ (i) have a local support, (ii) are positive on their supports and (iii) form a partition of unity. Every function s(t) satisfying Definition 1 then has a unique representation:

(2) $$s\left(t\right)=\sum_{i=1}^{z}c_{i}N_{i,r,\xi }\left(t\right)=n{\left(t\right)}^{T}c,$$

with $n\left(t\right)={\left[N_{1,r,\xi }\left(t\right),\ldots ,N_{z,r,\xi }\left(t\right)\right]}^{T}$, $c={\left[c_{1},\ldots ,c_{z}\right]}^{T}$, where $N_{i,r,\xi }\left(t\right)$ or shortly $N_{i}\left(t\right)$, i=1,…,z, denote base functions of the spline space $P_{r,\xi ,\alpha }$, and $c_{i}$ denotes the *i*-th B-spline coefficient of s(t). They are commonly referred to as control coefficients or control points, and the collection ${\left\{c_{i}\right\}}_{i=1}^{z}$ of all control points is referred to as the control polygon of the spline. In the following, we will assume the spline represented as a linear combination of basis B-spline functions (2) as the approximation function. The spline design parameters thus are the following:

- The order *r* and defect $d_{\mathrm{ef}}$ of the spline;
- The number *q* and location ξ of its knots;
- The control coefficients (control polygon) ${\left\{c_{i}\right\}}_{i=1}^{z}$.

### 2.2. Conversion from B- to pp-Representation

A polynomial spline can be, by definition, written as

(3) $$\begin{array}{ccc} s\left(t\right)=p_{i}\left(t\right):=\sum_{j=1}^{r}p_{ij}{\left(t-\xi_{i}\right)}^{j-1},\; & t\in [\xi_{i},\xi_{i+1}],\; & i=0,\ldots ,q, \end{array}$$

where $p_{i}\left(t\right)$ are polynomial pieces or segments which represent the spline s(t) on interval $\left[0,T_{x}\right]$. The relation (3) is called a piecewise polynomial (pp-)representation of the spline s(t). Clearly, the pp-representation of spline s(t) is completely determined by the (q+1)r-dimensional vector of the polynomial coefficients $p={\left[p_{01},\ldots ,p_{0r},\cdots ,p_{q1},\ldots ,p_{qr}\right]}^{T}$. Given the B-representation (2) of the spline s(t), the vector p of its pp-representation can be easily computed according to

(4) p=Pc,

where rows of matrix P can be obtained by the differentiation of vector $n^{T}\left(t\right)$ in knots ${\left\{\xi_{i}\right\}}_{i=1}^{q}$:

$$P={\left[\frac{1}{(j-1)!}{\left.{n^{\left[j-1\right]}}^{T}\left(t\right)\right|}_{t=\xi_{i}}\right]}_{j=1,\ldots ,r}^{i=0,\ldots ,q}$$

with

$${n^{\left[j-1\right]}}^{T}\left(t\right)=\left[N_{1}^{\left[j-1\right]}\left(t\right),\ldots ,N_{z}^{\left[j-1\right]}\left(t\right)\right],$$

where notation $f^{\left[i\right]}\left(t\right)$ stands for the *i*-th derivative of f(t) with respect to *t*, with $f^{\left[0\right]}\left(t\right)\equiv f\left(t\right)$; see [23,27] for details.

Given the vector p, the conversion from pp-representation to B-representation can be performed as follows:

$$c=P_{L}^{-1}p,$$

which is more difficult because of the left inverse of matrix P; however, if it is a priori known that the approximated function lies in $P_{r,\xi ,\alpha }$ for a certain r,ξ,α, then $P_{L}^{-1}$ can be determined uniquely.

### 2.3. Shape Properties of B-Spline Curves

The fundamental shape properties of B-spline curves that we rely on in this article may be summarized using the following theorem. The reader is referred to, e.g., [26] for more details and proofs.

**Theorem 1**  (Shape properties of B-spline curves). *Let s(t) be a B-spline curve of order r over the knot sequence ξ. Then, the following properties hold:*

(*i*)  *In general, there is no endpoint interpolation;*
(*ii*)  *For $\xi_{i}\leq t<\xi_{i+1}$, s(t) lies in the convex hull of the r control points $c_{i-r+1},\ldots ,c_{i}$;*
(*iii*)  *Local control: for $t\in [\xi_{i},\xi_{i}+1]$, the curve is independent of $c_{j}$ for j<i−r+1 and j>i;*
(*iv*)  *If r−1 control points coincide, then the spline curve passes through this point and is tangent to the control polygon;*
(*v*)  *If r−1 control points are on a line, then the spline curve touches this line;*
(*vi*)  *If r control points are on a line L, then s(t)∈L for $\xi_{i}\leq t<\xi_{i+1}$, i.e., an entire segment of the curve s(t) coincides with L;*
(*vii*)  *Its derivative is*
  $$s^{\prime }\left(t\right)=\sum_{i=1}^{z-1}{\bar{c}}_{i}N_{i,r-1,\xi }\left(t\right)\;\;\;\mathrm{with}\;\;\;{\bar{c}}_{i}=\frac{r-1}{\xi_{i+r-1}-\xi_{i}}\left(c_{i}-c_{i+1}\right);$$
(*viii*)  *If r−1 knots $t=\xi_{i+1}=\ldots =\xi_{i+r-1}$ coincide, then $s\left(t\right)=c_{i}$, i.e., the spline curve passes through a control point and is tangent to the control polygon.*

## 3. Problem Formulation

The problem formulation will be presented using the example of the aforementioned laboratory 2-DOF helicopter, the hardware of which will be described later in Section 4. In some passages, it will follow formulations presented by the authors in [17] which led to the explicit solution to the spline-based CMPC problem.

Let us in general consider the laboratory 2-DOF helicopter as a continuous-time MIMO system described near a given operating point by the following linear time-invariant state-space form (in reality, in our application, it will be a two-input two-output (TITO) system):

(5a) $$\begin{array}{cc} {\dot{x}}_{h}\left(t\right) & =A_{h}x_{h}\left(t\right)+B_{h}u\left(t\right), \end{array}$$

(5b) $$\begin{array}{cc} y(t) & =C_{h}x_{h}\left(t\right), \end{array}$$

where $A_{h}$, $B_{h}$ and $C_{h}$ are state-space matrices of appropriate dimensions and the elements of the state vector $x_{h}\left(t\right)\in R^{r_{u}}$ ($r_{u}$ denoted the order of the spline input signal) can be directly calculated from the spline derivatives of input/output signals $u\left(t\right)\in R^{n_{u}},y\left(t\right)\in R^{n_{y}}$ by using the multi-input single-output (MISO) technique formulated below.

### 3.1. Phase-Variable State-Space Description

Let us consider the MIMO system description (5) in the deterministic framework and apply it over the spline output and input signals, $s_{y_{i}}\left(t\right)$ and $s_{u_{j}}\left(t\right)$, $i=1\ldots n_{y},\;j=1\ldots n_{u}$. Then, for the *i*-th spline output signal, $s_{y_{i}}\left(t\right)$, of the system, the following MISO differential equation can be written:

(6) $$\begin{array}{cc} \; & s_{y_{i}}^{\left[\rho_{\alpha }\right]}\left(t\right)+\alpha_{i,1}s_{y_{i}}^{\left[\rho_{\alpha }-1\right]}\left(t\right)+\ldots +\alpha_{i,\rho_{\alpha }}s_{y_{i}}\left(t\right)=\\ \; & =\sum_{j=1}^{n_{u}}\left[\beta_{0_{i,j}}s_{u_{j}}^{\left[\rho_{\beta }\right]}\left(t\right)+\beta_{1_{i,j}}s_{u_{j}}^{\left[\rho_{\beta }-1\right]}\left(t\right)+\ldots +\beta_{\rho_{\beta_{i,j}}}s_{u_{j}}\left(t\right)\right],\;\rho_{\beta }\leq \rho_{\alpha }, \end{array}$$

where coefficients $\alpha_{i,k}$ and $\beta_{l_{i,j}}$, $k=1\ldots \rho_{\alpha },l=1\ldots \rho_{\beta }$, are real constant scalars. For the degrees $\rho_{\alpha }$ and $\rho_{\beta }$, it holds that $\rho_{\alpha }\leq \left(r_{y}-1\right)$ and $\rho_{\beta }\leq \left(r_{u}-1\right)$, respectively.

Having specified spline input–output derivatives, by applying a common technique of moving data windows it is easy to estimate the vector of unknown parameters of (6) through linear regression:

$$\begin{array}{cc} s_{y_{i}}^{\left[\rho_{\alpha }\right]}\left(t\right) & =\theta_{s}^{iT}\;\varphi_{s}^{i}\left(t\right))+\epsilon_{s}^{i}\left(t\right),\\ \theta_{s}^{i} & ={\left[\alpha_{i,1},\ldots \alpha_{i,\rho_{\alpha }},\beta_{0_{i,1}},\ldots \beta_{\rho_{\beta_{i,1}}}\cdots \beta_{0_{i,n_{u}}},\ldots \beta_{\rho_{\beta_{i,n_{u}}}}\right]}^{T},\\ \varphi_{s}^{i}\left(t\right) & ={\left[-s_{y_{i}}^{\left[\rho_{\alpha }-1\right]}\left(t\right),\ldots -s_{y_{i}}\left(t\right),s_{u_{1}}^{\left[\rho_{\beta }\right]}\left(t\right),\ldots s_{u_{1}}\left(t\right)\cdots s_{u_{n_{u}}}^{\left[\rho_{\beta }\right]}\left(t\right),\ldots s_{u_{n_{u}}}\left(t\right)\right]}^{T}, \end{array}$$

and a linear square approach where $\epsilon_{s}^{i}\left(t\right)$ denotes an equation error.

Several methods are available for finding possible state-space forms from the input–output map (6). The well-known phase-variable canonical forms, see, e.g., [28], are particularly useful for our purposes because state variables can be defined in terms of the plant variables and their derivatives. With this, we can choose the $\rho_{\alpha }$-dimensional state vector $x^{i}\left(t\right)={\left[x_{1}^{i}\left(t\right)\ldots x_{\rho_{\alpha }}^{i}\left(t\right)\right]}^{T}$, of a phase-variable canonical form related to the *i*-th spline output, as

(7) $$\begin{array}{c} x^{i}\left(t\right)=S_{y_{i}}\;{\bar{s}}_{y_{i[\rho_{\alpha }-1]}}\left(t\right)+\sum_{j=1}^{n_{u}}S_{u_{j}}^{i}\;{\bar{s}}_{u_{j[\rho_{\beta }-1]}}\left(t\right) \end{array}$$

with $\left(\rho_{\alpha }\times \rho_{\alpha }\right)$ square matrices $S_{y_{i}},S_{u_{i,j}}$ defined as

$$S_{y_{i}}=\left[\begin{array}{ccccc} 1 & \alpha_{i,1} & \alpha_{i,2} & \ldots & \alpha_{i,\rho_{\alpha }-1}\\ 0 & 1 & \alpha_{i,1} & \ldots & \alpha_{i,\rho_{\alpha }-2}\\ 0 & 0 & 1 & \ldots & \alpha_{i,\rho_{\alpha }-3}\\ \vdots & \vdots & \vdots & \ddots & \vdots \\ 0 & 0 & 0 & \ldots & 1 \end{array}\right],S_{u_{j}}^{i}=\left[\begin{array}{cccccc} 0 & \ldots & -\beta_{0_{i,j}} & -\beta_{1_{i,j}} & \ldots & -\beta_{\rho_{\beta }-1_{i,j}}\\ 0 & \ldots & 0 & -\beta_{0_{i,j}} & \ldots & -\beta_{\rho_{\beta }-2_{i,j}}\\ \vdots & \vdots & \vdots & \vdots & \ddots & \vdots \\ \vdots & \vdots & \vdots & \vdots & \vdots & -\beta_{0_{i,j}}\\ \vdots & \vdots & \vdots & \vdots & \vdots & \vdots \\ 0 & \ldots & 0 & 0 & \ldots & 0 \end{array}\right],$$

and with $\rho_{\alpha }$-dimensional vectors ${\bar{s}}_{y_{i[\rho_{\alpha }-1]}}\left(t\right)$, ${\bar{s}}_{u_{j[\rho_{\beta }-1]}}\left(t\right)$ containing spline derivatives of the *i*-th plant output and the *j*-th plant input in a backward location, compared with $s_{y_{i[\rho_{\alpha }-1]}}\left(t\right)$, $s_{u_{j[\rho_{\beta }-1]}}\left(t\right)$. In the case of ${\bar{s}}_{u_{j[\rho_{\beta }-1]}}\left(t\right)$, the missing entries to the dimension $\rho_{\alpha }$ are completed by a corresponding amount of zeros:

(8) $${\bar{s}}_{u_{j[\rho_{\beta }-1]}}\left(t\right)={\left[0,\ldots ,0,s_{u_{j}}^{\left[\rho_{\beta }-1\right]}\left(t\right),\ldots ,s_{u_{j}}^{\left[1\right]}\left(t\right),s_{u_{j}}\left(t\right)\right]}^{T}.$$

The foregoing equations can be arranged in the following MISO state-space form:

(9a) $$\begin{array}{cc} {\dot{x}}^{i}\left(t\right) & =A_{i}\;x^{i}\left(t\right)+B_{i}\;s_{u}\left(t\right), \end{array}$$

(9b) $$\begin{array}{cc} s_{y_{i}}\left(t\right) & =c\;x^{i}\left(t\right), \end{array}$$

where

$$\begin{array}{cc} A_{i} & =\left[\begin{array}{cccccc} 0 & 0 & 0 & \ldots & 0 & -\alpha_{i,\rho_{\alpha }}\\ 1 & 0 & 0 & \ldots & 0 & -\alpha_{i,\rho_{\alpha }-1}\\ 0 & 1 & 0 & \ldots & 0 & {}_{-}\alpha_{i,\rho_{\alpha }-2}\\ \vdots & \vdots & \vdots & \vdots & \vdots & \vdots \\ 0 & 0 & 0 & \ldots & 0 & -\alpha_{i,2}\\ 0 & 0 & 0 & \ldots & 1 & -\alpha_{i,1} \end{array}\right],\\ B_{i} & =\left[\begin{array}{ccc} \beta_{\rho_{\beta i,1}} & \ldots & \beta_{\rho_{\beta i,n_{u}}}\\ \vdots & \vdots & \vdots \\ \beta_{0_{i,1}} & \ldots & \beta_{0_{i,n_{u}}}\\ 0 & \ldots & 0\\ \vdots & \vdots & \vdots \\ 0 & \ldots & 0 \end{array}\right],\;\;s_{u}\left(t\right)=\left[\begin{array}{c} s_{u_{1}}\left(t\right)\\ \vdots \\ s_{u_{n_{u}}}\left(t\right) \end{array}\right],\\ c & =\left[\begin{array}{ccccc} 0 & 0 & \ldots & 0 & 1 \end{array}\right]. \end{array}$$

By assuming $i=1\ldots n_{y}$, the set of MISO realizations (9) can be used to create the following simple MIMO state-space realization of the system which is valid near the given operating point:

(10a) $$\begin{array}{cc} {\dot{x}}_{m}\left(t\right) & =A_{m}\;x_{m}\left(t\right)+B_{m}\;s_{u}\left(t\right), \end{array}$$

(10b) $$\begin{array}{cc} s_{y}\left(t\right) & =C_{m}\;x_{m}\left(t\right), \end{array}$$

with order $n=n_{y}.\rho_{\alpha }$, and the state and output vectors defined as

$$x_{m}\left(t\right)=\left[\begin{array}{c} x^{1}\left(t\right)\\ \vdots \\ x^{n_{y}}\left(t\right) \end{array}\right],\;\;s_{y}\left(t\right)=\left[\begin{array}{c} s_{y_{1}}\left(t\right)\\ \vdots \\ s_{y_{n_{y}}}\left(t\right) \end{array}\right].$$

It is easy to verify that

$$\begin{array}{c} A_{m}=\left[\begin{array}{cccc} A_{1} & & & \\ & A_{2} & & \\ & & \ddots & \\ & & & A_{n_{y}} \end{array}\right],\;\;B_{m}=\left[\begin{array}{c} B_{1}\\ B_{2}\\ \vdots \\ B_{n_{y}} \end{array}\right],\;\;C_{m}=\left[\begin{array}{cccc} c & & & \\ & c & & \\ & & \ddots & \\ & & & c \end{array}\right]. \end{array}$$

The realization (10) is a straightforward connection of canonical realizations (9) for each system output and therefore is not the minimal one. Nevertheless, in this realization, the *n*-dimensional state vector $x_{m}\left(t\right)$ is accessible to direct measurement.

If the system’s operating point varies in time—and this is our case—then the description (10) also becomes time-variant and can be written as:

(11a) $$\begin{array}{cc} {\dot{x}}_{m}\left(t\right) & =A_{m}\left(t\right)\;x_{m}\left(t\right)+B_{m}\left(t\right)\;s_{u}\left(t\right), \end{array}$$

(11b) $$\begin{array}{cc} s_{y}\left(t\right) & =C_{m}\left(t\right)\;x_{m}\left(t\right), \end{array}$$

where the matrices $A_{m}\left(t\right)$ and $B_{m}\left(t\right)$ can be easily identified online using, e.g., algorithms of the recursive least squares method; thus, the overall design of the control scheme will enter the adaptive mode.

### 3.2. B-Spline Parameterization of CMPC Formulation

In this subsection, we discuss the CMPC formulation from the viewpoint of the application of B-spline parameterization as a starting point for obtaining computationally efficient control algorithms. In order to be able to introduce the integral action in the resulting formulations, we are further more interested in the derivatives of control input signals contained in the vector $\dot{u}\left(\tau \right)$. Using the expansion (2) with the same choice of B-spline function parameters for all inputs (i.e., $u_{i}\left(\tau \right)\equiv s_{u_{i}}\left(\tau \right)=n{\left(\tau \right)}^{T}c_{u_{i}}$, $u_{i}\left(\tau \right)\in P_{r_{u},\xi ,\alpha },i=1,\ldots ,n_{u}$), these can be obtained as

(12) $$\dot{u}\left(\tau \right)=\dot{N}\left(\tau \right)c_{u},$$

where

$$\dot{N}\left(\tau \right)=\left[\begin{array}{ccc} {\dot{n}}^{T}\left(\tau \right) & & \\ & \ddots & \\ & & {\dot{n}}^{T}\left(\tau \right) \end{array}\right],\;\;c_{u}=\left[\begin{array}{c} c_{u}^{1}\\ \vdots \\ c_{u}^{n_{u}} \end{array}\right],$$

with $c_{u}^{i}={\left[c_{1}^{i},\ldots ,c_{z}^{i}\right]}^{T},i=1,\ldots ,n_{u}$ and $\dot{n}\left(\tau \right)$ denoting a vector of the first derivatives of the used B-spline functions.

By taking the derivative of both sides of (11) with $u\left(t\right)\equiv s_{u}\left(t\right)$ and $y\left(t\right)\approx s_{y}\left(t\right)$, the state-space model can be rewritten in the following augmented form:

(13a) $$\begin{array}{cc} \dot{x}\left(t\right) & =A\left(t\right)\;x\left(t\right)+B\left(t\right)\;\dot{u}\left(t\right), \end{array}$$

(13b) $$\begin{array}{cc} y(t) & =C\;x(t), \end{array}$$

where

$$\begin{array}{cc} x(t) & ={\left[\begin{array}{cc} z^{T}\left(t\right) & y^{T}\left(t\right) \end{array}\right]}^{T},\;\;z\left(t\right)={\dot{x}}_{m}\left(t\right),\\ A(t) & =\left[\begin{array}{cc} A_{m}\left(t\right) & 0\\ C_{m} & 0 \end{array}\right],\;\;B\left(t\right)=\left[\begin{array}{c} B_{m}\left(t\right)\\ 0 \end{array}\right],\;\;C=\left[\begin{array}{cc} 0 & I \end{array}\right]. \end{array}$$

Assuming a time window given by $\tau \in [t_{k},t_{k}+T_{h}]$, where $t_{k}$ is the current time and $T_{h}$ denotes the prediction horizon, the predicted state at time $t_{k}+\tau$ can be obtained by solving the differential Equation (13a) as follows:

(14) $$x\left(t_{k}+\tau \right)=e^{A_{k}\tau }x\left(t_{k}\right)+\int_{0}^{\tau }e^{A_{k}\left(\tau -\gamma \right)}B_{k}\dot{u}\left(t_{k}+\gamma \right)d\gamma ,\;$$

where for simplicity we used the notation $A_{k}=A\left(t_{k}\right)$ and $B_{k}=B\left(t_{k}\right)$. Now, if we employ the B-spline functions-based expansion (12) to approximate the control input derivatives, we can substitute them into the prediction Equation (14), which thus becomes parameterized in $c_{u}$:

(15) $$x\left(t_{k}+\tau \right)=e^{A_{k}\tau }x\left(t_{k}\right)+\Gamma_{k}\left(\tau \right)c_{u}\;$$

with $\Gamma_{k}\left(\tau \right)=\int_{0}^{\tau }e^{A_{k}\left(\tau -\gamma \right)}B_{k}\dot{N}\left(\gamma \right)d\gamma$.

The objective of the velocity-form CMPC design considered in this work is to drive the predicted system outputs, y(τ), as close as possible to predefined reference trajectories $y_{s}\left(\tau \right)$, ideally to solve the following infinite-horizon control problem:

(16a) $$\begin{array}{cc} \min_{u(\;\cdot \;)}\;\; & \int_{0}^{\infty }\left({∥y\left(\tau \right)-y_{s}\left(\tau \right)∥}_{Q_{y}\left(\tau \right)}^{2}+{∥\dot{u}\left(\tau \right)∥}_{Q_{u}\left(\tau \right)}^{2}\right)d\tau \end{array}$$

(16b) $$\begin{array}{cc} s.t.\; & u^{\min}\leq u\left(\tau \right)\leq u^{\max},\;\tau \geq 0, \end{array}$$

(16c) $$\begin{array}{cc} \; & {\dot{u}}^{\min}\leq \dot{u}\left(\tau \right)\leq {\dot{u}}^{\max},\;\tau \geq 0,\; \end{array}$$

where within the quadratic objective (16a), $Q_{y}\left(\tau \right)⪰0$ and $Q_{u}\left(\tau \right)⪰0$ denote the output and input weighting matrix, respectively. In the constraints (16b) and (16c), ($u^{\min},u^{\max}$) and $({\dot{u}}^{\min},{\dot{u}}^{\max}$) denote bounds imposed on control signals and their derivatives, respectively.

Now, applying the quasi-infinite horizon approach to guarantee the closed-loop stability, our goal is to solve the following finite-horizon continuous-time MPC problem:

(17a) $$\begin{array}{cc} \min_{u(\;\cdot \;)}\; & \int_{t_{k}}^{t_{k}+T_{h}}\left({∥x\left(t_{k}+\tau |t_{k}\right)-x_{s}\left(t_{k}+\tau |t_{k}\right)∥}_{Q_{x}\left(\tau \right)}^{2}+{∥\dot{u}\left(t_{k}+\tau |t_{k}\right)∥}_{Q_{u}\left(\tau \right)}^{2}\right)d\tau \\ \; & \;\;\;\;\;+{∥\tilde{x}\left(t_{k}+T_{h}|t_{k}\right)∥}_{Q_{h,k}}^{2}\; \end{array}$$

(17b) $$\begin{array}{cc} s.t.\; & u^{\min}\leq u\left(t+\tau |t\right)\leq u^{\max},\;\tau \geq t, \end{array}$$

(17c) $$\begin{array}{cc} \; & {\dot{u}}^{\min}\leq \dot{u}\left(t+\tau |t\right)\leq {\dot{u}}^{\max},\;\tau \geq t, \end{array}$$

(17d) $$\begin{array}{cc} \; & \;\;\;\tilde{x}\left(t_{k}+T_{h}|t_{k}\right)\in \Omega_{k},\; \end{array}$$

where $Q_{x}\left(\tau \right)=C^{T}Q_{y}\left(\tau \right)C$, $x_{s}\left(\tau \right)$ denotes the state reference trajectories defined by the setpoints, and $\tilde{x}\left(t_{k}+T_{h}|t_{k}\right)$ denotes a deviation from the steady-state target calculated for the current reference state $x_{s}\left(\tau \right)$ at $\tau =T_{h}$. In addition, the stability and recursive feasibility of the control problem are ensured by assuming a terminal cost weighted with $Q_{h,k}⪰0$ in (17a), and by assuming a terminal set $\Omega_{k}$ constraint (17d). This set has to be invariant under a local linear state feedback u=Fx, virtually acting for $\tau \in [t_{k}+T_{h},\infty [$ and feasible with (17b) and (17c). For the calculation of $\Omega_{k}$ and $Q_{h,k}$, a simple computational procedure proposed in [29] can be adopted.

The adaptive CMPC problem (17) can be tackled using B-spline parameterization which leads to the following formulation:

(18a) $$\begin{array}{cc} \min_{c_{u}\left(k\right)}\; & c_{u}{\left(k\right)}^{T}H_{k}c_{u}\left(k\right)+2c_{u}{\left(k\right)}^{T}\left(G_{k}x\left(t_{k}\right)-Q_{s,k}c_{s}\left(t_{k}\right)\right), \end{array}$$

(18b) $$\begin{array}{cc} s.t.\; & \;\;\;c_{u}^{\min}\leq c_{u}\left(k\right)\leq c_{u}^{\max}, \end{array}$$

(18c) $$\begin{array}{cc} \; & c_{u,\Delta }^{\min}\leq A_{\Delta }c_{u}\left(k\right)\leq c_{u,\Delta }^{\max}, \end{array}$$

(18d) $$\begin{array}{cc} \; & \;x_{\Omega_{k}}^{\min}\leq A_{\Omega_{k}}c_{u}\left(k\right)\leq x_{\Omega_{k}}^{\max}, \end{array}$$

(18e) $$\begin{array}{cc} \; & \;\;n{\left(0\right)}^{T}c_{u}\left(k\right)=n{\left(T_{h}\right)}^{T}c_{u}\left(k-1\right), \end{array}$$

(18f) $$\begin{array}{cc} \; & \;\;\dot{n}{\left(0\right)}^{T}c_{u}\left(k\right)=\dot{n}{\left(T_{h}\right)}^{T}c_{u}\left(k-1\right), \end{array}$$

(18g) $$\begin{array}{cc} \; & \;\;\;\;\dot{n}{\left(T_{h}\right)}^{T}c_{u}\left(k\right)=0. \end{array}$$

The reformulated objective (18a) was obtained by substituting (12) and (15) into (17a), while expressing the setpoints as spline functions using the relation (2) as follows:

$$y_{s}\left(t_{k}+\tau |t_{k}\right)=N_{s}\left(\tau \right)c_{s}\left(t_{k}\right),$$

where

$$N_{s}\left(\tau \right)=\left[\begin{array}{ccc} n_{s}^{T}\left(\tau \right) & \; & \\ \; & \ddots & \\ \; & \; & n_{s}^{T}\left(\tau \right) \end{array}\right],\;\;c_{s}\left(t_{k}\right)=\left[\begin{array}{c} c_{s}^{1}\left(t_{k}\right)\\ \vdots \\ c_{s}^{n_{y}}\left(t_{k}\right) \end{array}\right],$$

with $c_{s}^{i}\left(t_{k}\right)={\left[c_{s1}^{i}\left(t_{k}\right),\ldots ,c_{sz}^{i}\left(t_{k}\right)\right]}^{T},i=1,\ldots ,n_{y}$, and $c_{s}\left(t_{k}\right)$ is a vector of the splines’ control points expressing the trajectories of setpoints prescribed by the user for the desired system outputs’ behavior over the prediction horizon $\tau \in [t_{k},t_{k}+T_{h}]$. The matrices $H_{k}$, $G_{k}$ and $Q_{h,k}$ can be calculated, using numerical integration, as follows:

$$\begin{array}{cc} H_{k} & =\;\;\;\;\;\int_{t_{k}}^{t_{k}+T_{h}}\;\;\;\;\Gamma_{k}{\left(\tau \right)}^{T}Q_{x}\left(\tau \right)\Gamma_{k}\left(\tau \right)d\tau +\;\;\;\;\int_{t_{k}}^{t_{k}+T_{h}}\;\;\;\;N{\left(\tau \right)}^{T}Q_{u}\left(\tau \right)N\left(\tau \right)d\tau +\Gamma_{k}{\left(T_{h}\right)}^{T}Q_{h,k}\Gamma_{k}\left(T_{h}\right),\\ G_{k} & =\;\;\;\;\;\int_{t_{k}}^{t_{k}+T_{h}}\;\;\;\;\Gamma_{k}{\left(\tau \right)}^{T}C^{T}Q_{y}\left(\tau \right)Ce^{A_{k}\tau }d\tau +\Gamma_{k}{\left(T_{h}\right)}^{T}Q_{h,k}e^{A_{k}T_{h}},\;\;Q_{s,k}=\;\;\;\;\;\int_{t_{k}}^{t_{k}+T_{h}}\;\;\;\;\Gamma_{k}{\left(\tau \right)}^{T}C^{T}Q_{y}\left(\tau \right)N_{s}\left(\tau \right)d\tau . \end{array}$$

Note that the continuous-time constraints (17b) and (17c) had to be reformulated to suitable finite-dimensional forms given by (18b) and (18c), respectively. These must guarantee the intersample behavior of the spline control signal, which is achieved by a proper bounding of its control polygon $c_{u}\left(k\right)$. Taking into account the basic shape properties of B-splines, (18b) represents the amplitude constraints (17b) of the control signal u(t) imposed over a horizon $\left[t_{k},t_{k}+T_{h}\right]$, where $c_{u}^{\min}$ and $c_{u}^{\max}$ are the min and max values of the spline control coefficients $c_{u}\left(k\right)$ computed using the B-spline approximation of the given $u^{\min}$ and $u^{\max}$ values. Similarly, (18c) approximates the constraints (17c) on the derivative of the control signal $\dot{u}\left(t\right)$, where $c_{u,\Delta }^{\min}$ and $c_{u,\Delta }^{\max}$ are the min and max values of the differences in the spline control coefficients, which can, together with entries of matrix $A_{\Delta }$, be computed based on the location of the spline knots. Next, (18d) represents the terminal constraints transformed from (17d) using the prediction model (15), where $x_{\Omega_{k}}^{\min}$, $x_{\Omega_{k}}^{\max}$ denote the vectors bounding the terminal set $\Omega_{k}$. In order to keep the spline function $s_{u_{i}}\left(t\right)$ in the space $P_{r_{u},\xi ,\alpha }$ for the given ($r_{u},\xi ,\alpha$), the equality constraints (18e) and (18f) are used to enforce continuity between the implemented and the projected spline control signals at the beginning of the prediction horizon $\left[t_{k},t_{k}+T_{h}\right]$. Finally, the constraint (18g) is added to improve the stability of the control signals by requiring a zero derivative of the projected spline control signal at the end of the prediction horizon $\left[t_{k},t_{k}+T_{h}\right]$.

From the optimization perspective, the adaptive spline-based stabilizing CMPC problem in velocity form, (18), is a quadratic program (QP), which can be recast in the following simplified form:

(19a) $$\begin{array}{cc} \min_{c_{u}\left(k\right)}\; & \frac{1}{2}c_{u}{\left(k\right)}^{T}H_{k}c_{u}\left(k\right)+c_{u}{\left(k\right)}^{T}\left(G_{k}x\left(t_{k}\right)-Q_{s,k}c_{s}\left(t_{k}\right)\right), \end{array}$$

(19b) $$\begin{array}{cc} s.t.\; & A_{\mathrm{ineq}}c_{u}\left(k\right)\leq b_{\mathrm{ineq}}, \end{array}$$

(19c) $$\begin{array}{cc} \; & \;\;\;A_{\mathrm{eq}}c_{u}\left(k\right)=b_{\mathrm{eq}}. \end{array}$$

Solving QP (19) implicitly for a current state $x(t_{k})$ and given setpoints $c_{s}\left(t_{k}\right)$ yields a vector of optimal control coefficients $c_{u}^{★}\left(k\right)={\left[c_{1}^{★T},\ldots ,c_{n_{u}}^{★T}\right]}^{T}$, $c_{i}^{★}\in R^{z},i=1,\ldots ,n_{u}$. According to [15,16], $c_{u}^{★}\left(k\right)$ is subsequently converted to piecewise polynomial (pp)-representation of the individual optimal spline control signals $s_{u_{i}}^{★}\left(\tau \right)$, $\tau \in [t_{k},t_{k}+T_{h}]$, $i=1,\ldots ,n_{u}$ given by their polynomial coefficients contained in a vector $p^{★}\left(k\right)=P\;c_{u}^{★}\left(k\right)$.

**Figure 1:**
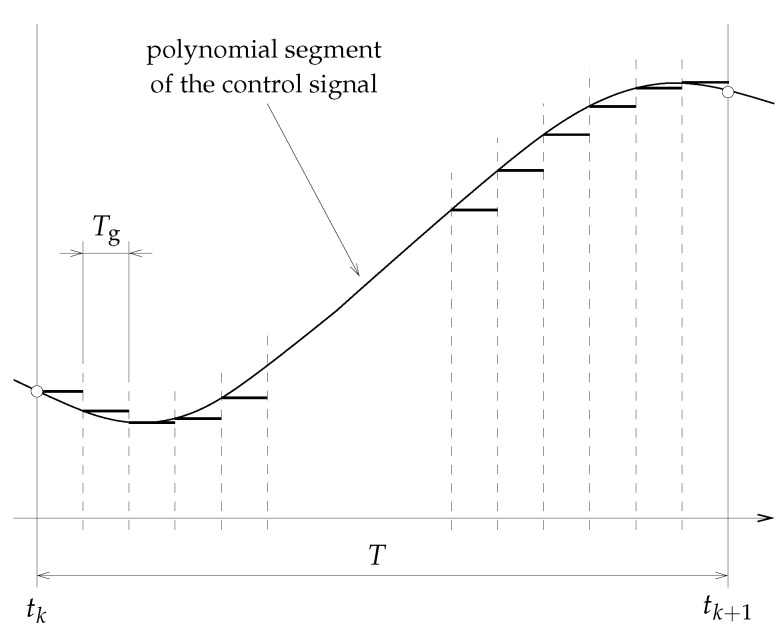
Illustration of how a spline control signal is generated. ∘ denotes a knot point, *T*—sampling period, $T_{g}$—implementation (generation) period.

The optimization problem (18) implies that we are looking at the control signals $u_{i}\left(t\right)$ from the viewpoint of a selected distance *T* between the knot points of the spline function $s_{u_{i}}\left(t\right)$. In real-time implementation, the distance *T* corresponds to the control period, in which the first polynomial segments of the respective optimal spline input signals, i.e., $s_{u_{i}}^{★}\left(\tau \right)$, $\tau \in [t_{k},t_{k}+T]$, are applied for control. This is usually performed with a much shorter implementation period $T_{g}\ll T$; see Figure 1 for illustration. This means that we calculate the values of the control signal $s_{u_{i}}^{★}\left(t_{k}+\tau \right)$ for the relative time variable $\tau =jT_{g}$, $j=1,\ldots ,n_{g}$, $T=n_{g}T_{g}$, $i=1,\ldots ,n_{u}$ and implement them for control using a common zero-order hold. The parameter $n_{g}$ is user-defined and its choice is important because it determines the degree of continuity of the generated signal.

### 3.3. Number of Polynomial Segments of Projected Spline Control Signals

Note that during the prediction horizon $\left[t_{k},t_{k}+T_{h}\right]$ the distance *T* between the knots of the projected spline input signal $s_{u_{i}}\left(\tau \right)$, $\tau \in [t_{k},t_{k}+T_{h}]$ can be chosen as βT, β=1,2,…, introducing a property comparable with the well-known move-blocking techniques used in MPC.

This is an important design parameter which—in conjunction with spline continuity conditions and the spline defect—allows us to tune various controller setups. Essentially, the number *q* of the polynomial segments in the projected spline control signal determines the degrees of freedom for a given task. A higher value of *q* provides a more active control signal, while a lower value leads to control which is more smooth and sluggish. Note that small values of q,q≥1 significantly reduce the QP computational effort.

The projected polynomial segments are interconnected in the interior knot points of the sequence $\xi_{h}$ which spans over the prediction horizon, $\left[t_{k},t_{k}+T_{h}\right]$. In general, the placement of these knots may be considered as another design tool. As mentioned earlier, we prefer a uniform location of these knots with a selected distance $T_{u}$, taken as $T_{u}=\beta T$. The distance then becomes a remarkable tuning knob for setting the overall length $T_{h}$ of the prediction horizon under the same number *q* of its segments. Using the distance $T_{u}$ as a variable, we can extend or shorten the prediction horizon satisfying the same computational burden due to the same dimension of the decision vector $c_{u}$. The chosen length of $T_{u}$ considerably dominates the activity of the projected spline control signal. Consequently, there is no need for any control weighting in the cost and it is possible to set $w_{u}\left(t\right)=0$, even for control of non-minimum phase plants. The setting $w_{u}\left(t\right)=0$ was assumed in all the experiments reported in this work.

**Figure 2:**
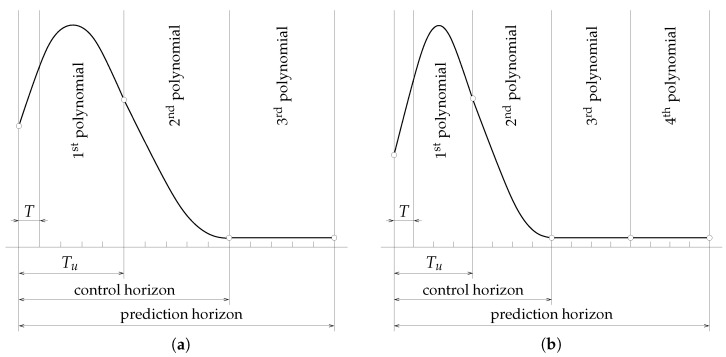
Illustration of a projected spline control signal with (**a**) 3 and (**b**) 4 polynomial segments.

One may tune different controllers via alternating continuity conditions and the spline defect in selected knots of the projected spline control signal. For example, it is possible to make—like in generalized predictive control—an assumption of zero increments of the projected control signal, starting from a certain time instant in the horizon, and thereby to impose an actual control horizon within the given prediction horizon. This can be simply achieved by constraining the knot derivatives of a certain number of last polynomial segments to zero while setting spline defects in the involved joining knots to zero as well. Applying the above procedure to a chosen number of segments, we can set a proper length of the control horizon, see, e.g., Figure 2a,b. For illustrative purposes, in these figures, two arrangements of third-order polynomial spline segments within the prediction horizon are presented. In Figure 2a, the derivatives of the last segment are set to zero, while in a similar arrangement in Figure 2b, a zero defect in the next-to-last knot is added. The impact on the actual control horizon length is evident.

Following the receding horizon strategy, only that piece of the first polynomial segment of the spline control signal which corresponds to the first sampling period *T* is really applied to the controlled system, as per Figure 1.

Overall, the proposed adaptive B-spline-based stabilizing CMPC algorithm boils down to performing the following steps at each time instant $t_{k}$:

1. For current plant data $y_{k}$, $c_{u}\left(k\right)$, and reference values $c_{s}\left(t_{k}\right)$, using a recursive least squares algorithm update the model (10) and calculate the state vector $x(t_{k})$; use it to
2. adapt the calculation of bounds $x_{\Omega_{k}}^{\min}$ and $x_{\Omega_{k}}^{\max}$ of the terminal set $\Omega_{k}$, and terminal cost $Q_{h,k}$; and to
3. update the matrices $H_{k}$, $G_{k}$ and $Q_{s,k}$;
4. solve the optimal control problem (18) as the QP (19) to obtain the vector of optimal control coefficients $c_{u}^{★}\left(k\right)$; which is then used to
5. calculate the vector $p^{★}\left(k\right)$ containing polynomial coefficients of all segments, using the relation (4) as $p^{★}\left(k\right)=P\;c_{u}^{★}\left(k\right)$; and
6. following the receding horizon strategy apply with the possibly shortest implementation period $T_{g},T_{g}\ll T$ the first polynomial segments of $p^{★}\left(k\right)$ to control the system during the time interval $\left[t_{k},t_{k}+T\right]$, which means calculate the control signals $s_{u_{i}}^{★}\left(t_{k}+\tau \right)$ for a relative time variable $\tau =jT_{g}$, $j=1,\ldots ,n_{g}$, $T=n_{g}T_{g}$, $i=1,\ldots ,n_{u}$, as spline polynomial segments of order $r_{u}$, and implement them for control using a common zero-order hold; and finally
7. repeat the procedure from step 1. for the next sampling instant $t_{k+1}=t_{k}+T$.

**Figure 3:**
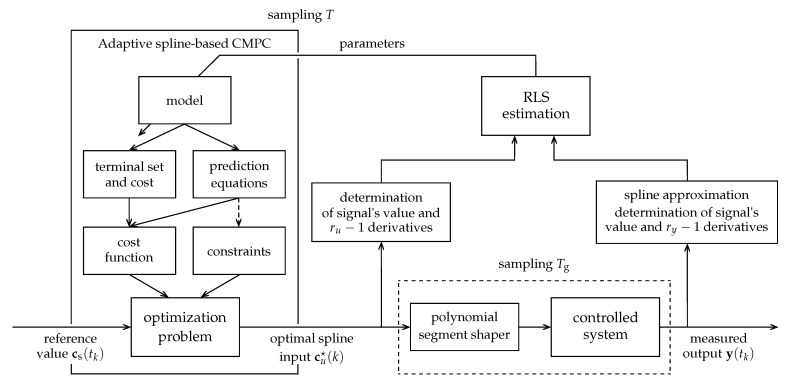
A simplified block scheme of the adaptive spline-based stabilizing CMPC feedback loop.

Figure 3 shows a simplified block scheme of the above algorithm.

## 4. Experimental Results and Discussion

This section describes the experiments carried out to verify the functionality and efficiency of the proposed concept of the B-spline CMPC algorithm. In the following subsections, we briefly explain the experimental 2-DOF helicopter platform and software tools, and after that, we illustrate some of the obtained results on four examples of tracking the predefined reference trajectories for the pitch and yaw angles. Similar platforms are well-known in the literature and are commonly used as benchmarks for testing various control techniques. In our case, the platform allows to easily demonstrate the basic features of the design and performance of the proposed B-spline-based CMPC controller.

### 4.1. Experimental Setup

**Figure 4:**
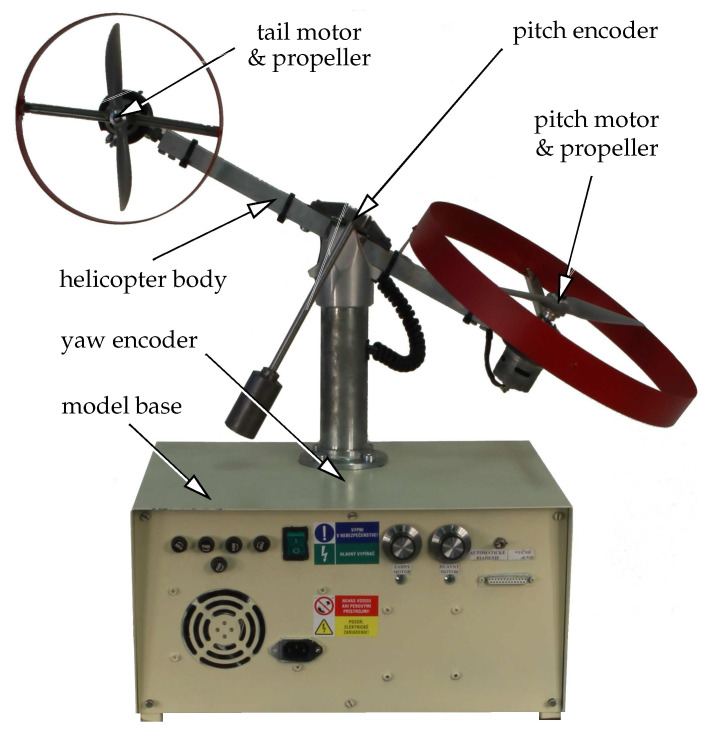
Photograph of the laboratory 2-DOF helicopter setup.

Our laboratory helicopter physical model [7] consists of a base and a beam carrying at its ends two propellers—the main and the tail one—driven by DC motors; see Figure 4. The beam is attached to its base via an articulated joint which allows the beam to rotate so that its ends move on spherical surfaces. This connection enables two degrees of freedom of the helicopter body movement—rotation around the horizontal axis, described by the pitch angle, and rotation around the vertical axis, described by the yaw angle. Both angles are measured using high-resolution incremental encoders. The model was designed at the authors’ workplace and represents a low-cost alternative to the commercially available models, such as [30], except for its limited rotation around the vertical axis imposed by power and data cables.

The axes of the main and the tail propellers as well as the vertical and the horizontal helicopter axes are usually perpendicular to each other so that the movement in the vertical plane and the movement in the horizontal plane are each affected by the thrust of only one propeller. A counter-weight fixed to the beam determines a stable equilibrium position. The system is balanced in such a way that when the motors are not powered, the main propeller end of the beam is lowered. As is usual for similar small-scale laboratory helicopter setups, the control is achieved exclusively by controlling the speeds of the propellers at a fixed angle of attack. The range of the helicopter body rotation is ± 30${}^{\circ }$ in the pitch angle and ± 145${}^{\circ }$ in the yaw angle. The plane of our main propeller is also slightly (∼ 2${}^{\circ }$) deviated from the horizontal one to strengthen the coupling effect with the tail propeller. The main source of random uncertainties in our laboratory helicopter design is the persistent vibration of the tail endpoint of the beam, which manifests itself in the increased fluctuation of the measured value of the helicopter yaw angle during the experiments.

The described laboratory helicopter model can be represented as a nonlinear multivariable system with two inputs:

- $u_{1}$—voltage driving propeller speed of the main motor;
- $u_{2}$—voltage driving propeller speed of the tail motor.

These are manipulated in the interface range of 0 V to 10 V. There are also two outputs:

- $y_{1}$—pitch (elevation) angle;
- $y_{2}$—yaw (azimuth) angle.

These are measured in degrees.

The interface voltages $u_{1}$ and $u_{2}$ applied for setting the helicopter inputs are converted to appropriate voltage values that drive the propeller motors. The output $y_{1}$ denotes the pitch angle in the vertical plane between the longitudinal axis of the helicopter body and the horizontal axis, and the output $y_{2}$ denotes the yaw angle in the horizontal plane between the longitudinal axis of the helicopter body and its zero (initial) position.

The voltage driving the main motor and the voltage driving the tail motor affect both the pitch angle and the yaw angle; hence, we can say that the interaction makes the system multivariable. It is also worth mentioning that the used mechanical simplification with fixed-angle propeller blades does not necessarily translate into simplified dynamics. On the contrary, the input torques and forces are applied via aerodynamical effects, as well as additional coupling effects appearing between the helicopter body and propellers dynamics, due to the reaction forces and torques arising at the acceleration or deceleration of the propellers. These increased cross-coupling effects have important implications on the control of the helicopter system and make its dynamics, as already mentioned, partially uncertain and time-variable. This is the reason why we consider the design of the control in an adaptive mode with RLS estimation of the model parameters and the adaptation of the calculation of the stabilizing terminal set. To solve the problem of RLS estimator wind-up, the estimator with directional forgetting from [21] was implemented. This estimator ensures the convergence of the estimation and avoids large changes in the model parameters.

The proposed spline-based CMPC scheme to control the laboratory helicopter shown in Figure 4 was implemented using the MATLAB/Simulink Desktop Real-Time prototyping suite on a mini PC equipped with a 2.8 GHz CPU and 16 G of RAM. Communication with the helicopter testbed was handled using the Humusoft’s MF644 multifunction desktop I/O card. The underlying QP (19) of the MPC problem is solved repeatedly at each control instant *T* using a parametric active-set algorithm implemented in the open-source software package qpOASES [31].

### 4.2. Experimental Results

In order to demonstrate the functionality and computational efficiency of the proposed spline-based CMPC scheme, we present four experiments assuming values of the design parameters listed in Table 1. These parameters are complemented by the selection of weighing matrices in the objective function (18), which were set as follows:

- As outlined, the input weighing matrix $Q_{u}$ was set as zero for all experiments.
- The output weighing matrix $Q_{y}$ was chosen as an exponential type with different exponential factors, $\lambda_{1}=0.995$ for the pitch angle and $\lambda_{2}=0.967$ for the yaw angle.

The parameters of the adaptive directional forgetting used in the experiments were chosen as follows:

- The minimum value of the adaptive forgetting factor was set to 0.97.
- The expected value of the adaptive forgetting factor in a steady state was set to 0.99.

All four controllers were tested to follow the same sequence of nonsimultaneous step changes in the pitch angle and yaw angle references. The experiments were conducted for a selection of two different implementation periods, namely $T_{g}=0.04\;s$ (experiments E1 and E2) and $T_{g}=0.02\;s$ (experiments E3 and E4). In doing so, the chosen setting of the quality parameters of the B-spline representation (spline orders, number and location of interior knots) in these experiments led to the number $n_{u}\left(r_{u}+q\right)$ of decision variables (i.e., the length of the optimizer vector) varying in the range of 10 to 14, as listed in Table 1.

**Figure 5:**
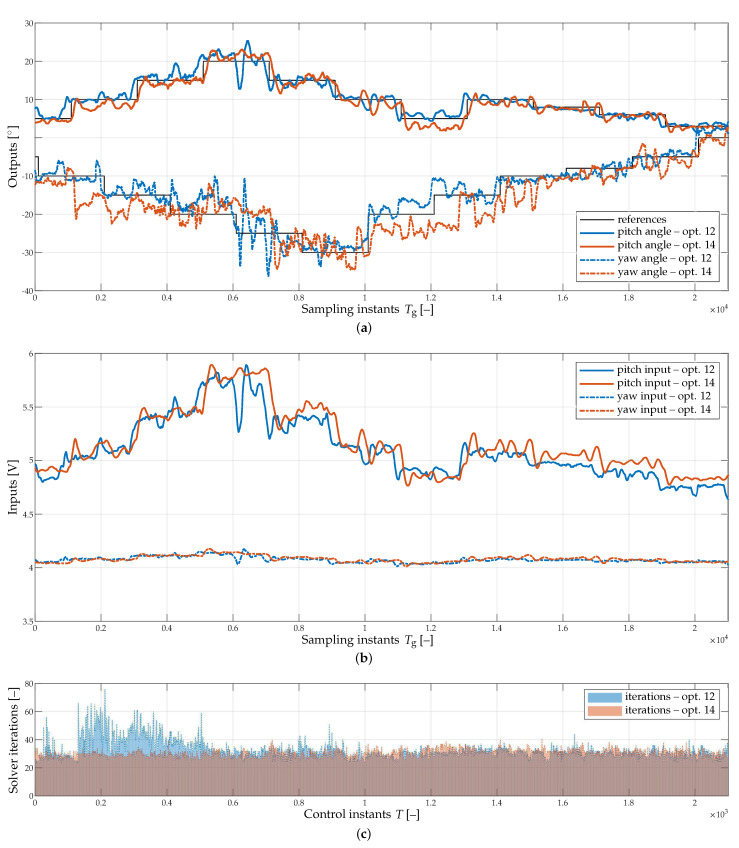
Experimental results of spline-based CMPC, compared for optimizer lengths 12 (experiment E1) and 14 (experiment E2), assuming $T_{g}=0.04\;s$. (**a**) Tracking of reference trajectories for pitch and yaw angles. (**b**) Voltage inputs applied to the helicopter rotors. (**c**) QP solver iterations.

The obtained tracking results for experiments E1 and E2 are presented in Figure 5a. One may notice the quite expected consequence that increasing the length of the optimizer improves the overall tracking performance, which is also evidenced by means of standard deviations of the tracking errors listed in Table 2. The comparison of the experiments is also supplemented by the profiles of the applied spline input signals, see Figure 5b, respecting the constraints imposed on the amplitude and the derivative. Finally, Figure 5c shows the numbers of iterations of the employed QP solver. Its addition is merely illustrative but to an extent confirms that lengthening the optimizer leads (under the same conditions) to lower numbers of required iterations.

**Figure 6:**
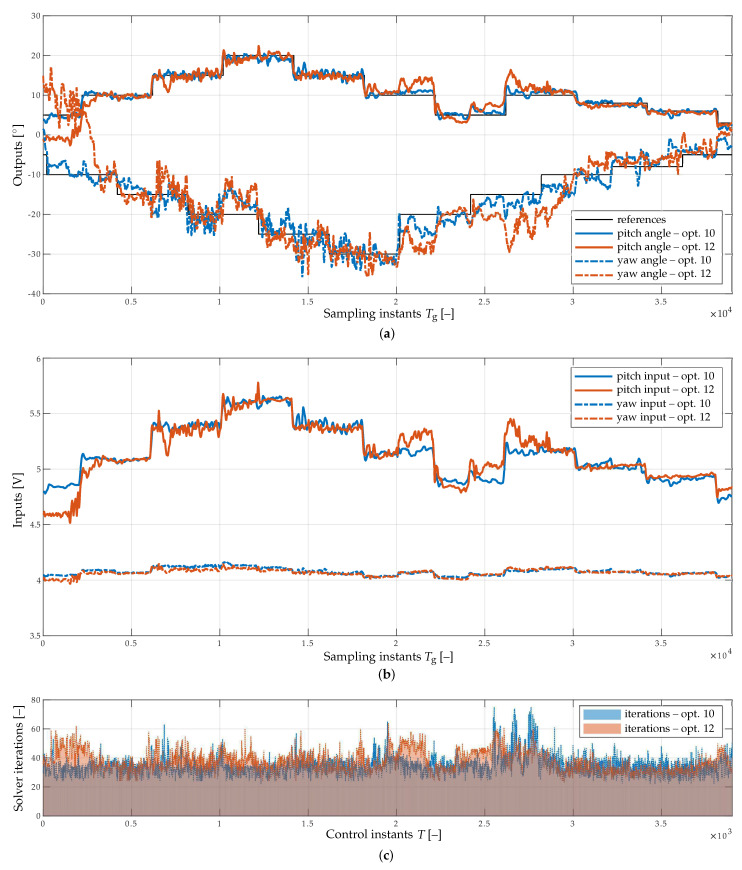
Experimental results of spline-based CMPC, compared for optimizer lengths 10 (experiment E3) and 12 (experiment E4), assuming $T_{g}=0.02\;s$. (**a**) Tracking of reference trajectories for pitch and yaw angles. (**b**) Voltage inputs applied to the helicopter rotors. (**c**) QP solver iterations.

The experiments were repeated with the implementation period $T_{g}=0.02\;s$ (experiments E3 and E4). As evidenced by Figure 6a and Table 1, the shorter implementation period contributed to an increase in the quality of tracking. Figure 6b,c illustrate the corresponding profiles of the spline input signals and solver iterations, respectively.

We can also observe from the performed experiments that the quality of tracking the reference trajectories was significantly affected by the existing cross-couplings, characteristic for the controlled helicopter system. This is owed mainly to the mutual link between the pitch angle and the yaw angle, which in this state of the solution we tried to suppress by the mentioned selection of the output weighing matrix $Q_{y}$.

We also remark that the zero input weighing matrix sufficed to ensure a stabilizing control. This observation is a by-product of the conditions (18e)–(18g) imposed on the continuity and zero derivative of the splines at the end of the prediction horizon.

In terms of evaluating the computational complexity, the main intention of this article is not to compare the proposed approach with other MPC formulations. Nevertheless, if we want to assess the computational burden of the proposed B-spline-based CMPC procedure compared with the standard discrete-time MPC procedure virtually acting on the same prediction horizon, we can approach it by comparing the sizes of the parameterizations that would be required by these procedures within the implementation, see Table 3, Table 4 and Table 5. They show that the B-spline parameterization is markedly leaner, i.e., computationally more efficient than the parameterization of a comparable standard discrete-time formulation, either in the case of parameterization assuming faster sampling given by control period $T_{g}$ (Table 5) or slower sampling given by *T* (Table 4). Regarding these comparisons, we may also point out that the CMPC procedure in addition enables to guarantee the intersample behavior—a property that may be approximated by the discrete-time formulation with shorter sampling time $T_{g}$, presented in Table 5.

## 5. Conclusions

This paper presented a numerically efficient approach to the synthesis of an adaptive velocity integration version of a continuous-time MPC based on B-spline parameterization. The design procedure is shown for multivariate systems represented by a set of MISO models. The implementation itself was carried out in an adaptive mode, with the adaptation of the model and the stabilizing terminal set, in order to improve the tracking capabilities of the controlled system in an uncertain and stochastic environment.

The resulting control strategy was applied for the pitch and yaw angle control of a 2-DOF laboratory helicopter. The performed real-time experiments demonstrated that the proposed B-spline CMPC by design represents a computationally viable alternative to the standard discrete-time MPC implementation. The obtained results are documented both graphically and numerically by capturing the standard deviations of the control errors. At the same time, they allow the reader or user to clearly understand the impact of optional parameters of the B-spline parameterization on the resulting tracking performance.

Finally, we remark that the proposed algorithm is typically coded on a single processor, although a dual-processor implementation would enable to separate the generation of the input polynomial segment from the optimization process, which could further contribute to the reduction in the tracking error.

## Figures and Tables

**Table 1:** Values of parameters used in real-time experiments.

Parameters	Experiments			
	E1	E2	E3	E4
Implementation period ${}^{†}$ $T_{g}$	0.04 s	0.04 s	0.02 s	0.02 s
Parameter $n_{g}$	10	10	10	10
Control period $T=n_{g}T_{g}$	0.4 s	0.4 s	0.2 s	0.2 s
Prediction horizon $T_{h}$	20 T	30 T	15 T	20 T
Order of spline input signals $r_{u}$	3	3	3	3
Order of spline output signals $r_{y}$	4	4	4	4
Order of spline reference signals $r_{s}$	4	4	4	4
Defect of splines $d_{\mathrm{ef}}$	1	1	1	1
Number *q* of interior knots within $T_{h}$	3	4	2	3
Location of interior knots within $T_{h}$	{5 T,10 T,15 T}	{6 T,12 T,18 T,24 T}	{5 T,10 T}	{5 T,10 T,15 T}
Move-blocking parameter β	5	6	5	5
Number of decision variables $n_{u}\left(r_{u}+q\right)$	12	14	10	12
Input amplitude bounds for main rotor	$u_{1}^{\min}=4.6$, $u_{1}^{\max}=5.7$	*★*	*★*	*★*
Input amplitude bounds for tail rotor	$u_{2}^{\min}=4.0$, $u_{2}^{\max}=4.2$	*★*	*★*	*★*
Input derivative bounds for main rotor	${\dot{u}}_{1}^{\min}=-1.9$, ${\dot{u}}_{1}^{\max}=1.9$	*★*	*★*	*★*
Input derivative bounds for tail rotor	${\dot{u}}_{2}^{\min}=-1.3$, ${\dot{u}}_{2}^{\max}=1.3$	*★*	*★*	*★*

${}^{†}$ Corresponds to sampling time. *★* Same as in experiment E1.

**Table 2:** Standard deviations of pitch and yaw control errors as attained during the experiments.

Experiment	Pitch Angle	Yaw Angle
E1	1.4870	3.3289
E2	1.2877	3.2910
E3	0.9294	3.1678
E4	0.8111	2.9105

**Table 3:** Size of B-spline parameterization of the CMPC assumed in real-time experiments E1 to E4.

Experiment	Prediction Horizon $T_{h}=\mathrm{NT}$ ${}^{†}$	Number of Decision Variables $n_{u}\left(r_{u}+q\right)$	Number of Input Amplitude and Derivative Constraints $4n_{u}\left(r_{u}+q\right)-2n_{u}$
E1	20 T	12	44
E2	30 T	14	52
E3	15 T	10	36
E4	20 T	12	44

${}^{†}$*N* denotes the number of prediction horizon steps.

**Table 4:** Size of parameterization of a comparable standard discrete-time MPC formulation virtually acting on prediction horizon $T_{h}$ with control period (sampling time) *T*.

Experiment	Prediction Horizon $T_{h}=\mathrm{NT}$	Number of Decision Variables $n_{u}N$	Number of Input Amplitude and Increment Constraints $n_{u}4N$
E1	20 T	40	160
E2	30 T	60	240
E3	15 T	30	120
E4	20 T	40	160

**Table 5:** Size of parameterization of a comparable standard discrete-time MPC formulation virtually acting on prediction horizon $T_{h}$ with control period (sampling time) $T_{g}$.

Experiment	Prediction Horizon $T_{h}={\mathrm{Nn}}_{g}T_{g}$	Number of Decision Variables $n_{u}{\mathrm{Nn}}_{g}$	Number of Input Amplitude and Increment Constraints $n_{u}4{\mathrm{Nn}}_{g}$
E1	200$T_{g}$	400	1600
E2	300$T_{g}$	600	2400
E3	150$T_{g}$	300	1200
E4	200$T_{g}$	400	1600

## Data Availability

The data presented in this study are available on request from the corresponding author. The data are not publicly available due to privacy.
